# Custom-Made Bifurcated Prosthetic Graft for Aortoiliac Aneurysm Repair

**DOI:** 10.1055/s-0041-1725090

**Published:** 2021-10-07

**Authors:** Spyros Papadoulas, Stavros K. Kakkos, Ioannis Ntouvas, Konstantinos Nikolakopoulos, Polyzois Tsantrizos, Chrysanthi Papageorgopoulou, Natasa Kouri

**Affiliations:** 1 Department of Vascular Surgery, University Hospital of Patras, Patras, Greece

**Keywords:** abdominal aortic aneurysm, iliac aneurysm, internal iliac revascularization, open repair, technique

## Abstract

Revascularization of the internal iliac artery during open repair of aortoiliac aneurysms can be challenging, especially if there is a significant distance between the orifices of the internal and external iliac arteries owing to common iliac aneurysmal dilatation. We describe a technique involving insertion of an 18-mm tube graft between the proximal aortic neck and aneurysmal common iliac artery bifurcation. Revascularization of the contralateral external iliac artery is accomplished through an 8-mm side arm graft.

## Introduction

Despite the evolution of endovascular techniques, repair of aortoiliac aneurysms is still performed by open surgery in a significant number of patients, particularly those with prolonged expected survival. Significant aneurysmal degeneration of the distal common iliac artery may lead to displacement of the orifices of the external and internal iliac arteries (EIA and IIA, respectively), making a formal end-to-end anastomosis with the graft limb of a bifurcated graft unsafe or even impossible. Several modifications of endovascular and open repair techniques have been described in the literature devised to overcome this problem. We describe an additional modification of the open reconstruction.

## Technique and Results

**Fig. 1:**
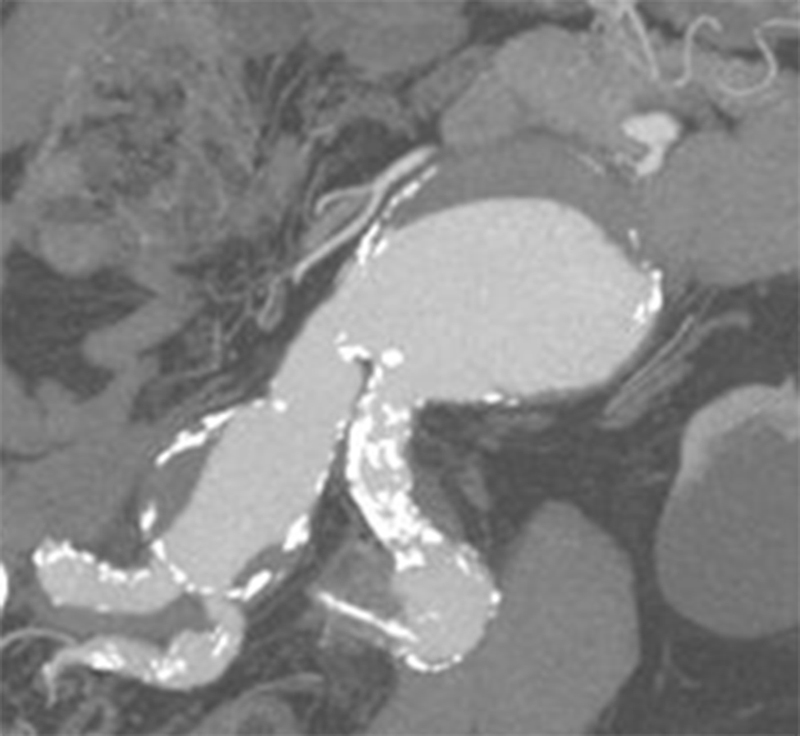
Computed tomography angiography showing an abdominal aortic aneurysm of 8 cm in diameter extending in both iliac arteries.

A 79-year-old male with lower abdominal pain radiating to the lumbar spine was admitted to the hospital and underwent a computed tomography angiography that revealed an abdominal aortic aneurysm (AAA) measuring 8 cm in diameter and extending to both EIA and IIA. The infrarenal neck measured 2.8 cm in diameter. Right and left common iliac aneurysms measured 4.2 and 3.8 cm in diameter, respectively ( Fig. 1 ). The right common iliac bifurcation measured 2.7 cm in diameter, while the distance between the origins of the EIA and IIA was 0.5 cm. Past medical history was significant for coronary artery disease, a 4-cm synchronous isolated descending thoracic aorta aneurysm, stroke without residual neurological deficit, and left inguinal hernia. During laparotomy, an intact aneurysm was found that was repaired with the technique described hereafter.

**Fig. 2:**
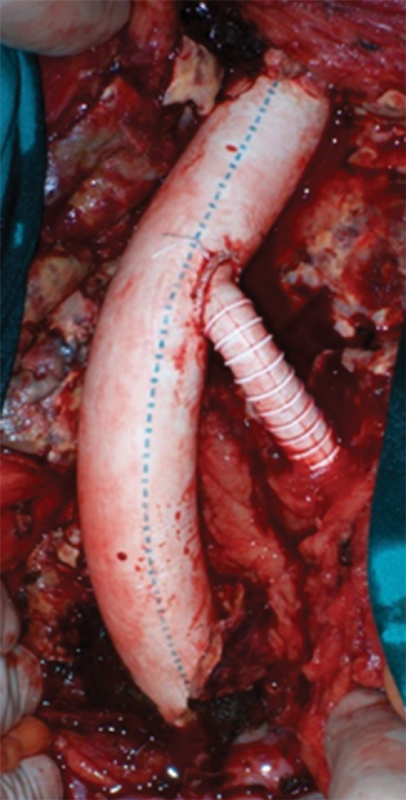
Intraoperative photo after the reconstruction.

**Fig. 3:**
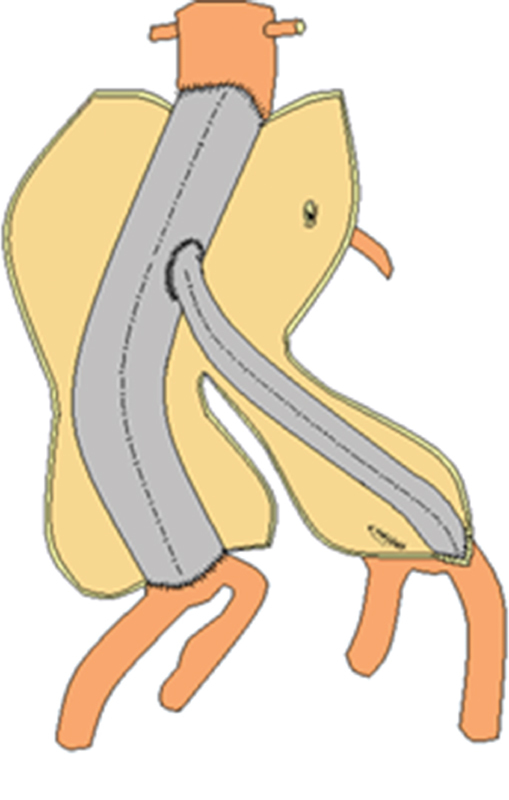
A drawing depicting the reconstruction.

The patient was operated under general orotracheal anesthesia in the supine position. Through a standard midline laparotomy, the aortic neck and the iliac bifurcations were dissected out in the usual fashion. The AAA was opened longitudinally and an 18-mm aortic tube graft (PFTE, Goretex Vascular Graft, W. L. Gore and Associates Inc., Flagstaff, AZ) was interposed. Proximally, the graft was sutured in an end-to-end fashion with the infrarenal aortic neck, and it was anastomosed in an end-to-end fashion with the right iliac bifurcation distally, encompassing both the orifices of the right IIA and right EIA. A separate side arm/tube graft (8 mm, Goretex Vascular Graft, W. L. Gore and Associates Inc.) was then anastomosed proximally with the aortic graft (in end-to-side fashion) and distally with the left EIA (in end-to-end fashion), while the left IIA was ligated ( Figs. 2 and 3 ). Postoperative course was uneventful, and the patient left the hospital on the seventh postoperative day. He did not develop buttock claudication on the left side as a result of IIA ligation; he passed away 3 years later after a mechanical fall in his home and severe depression, which left him bedridden for about a month.

## Discussion

Open repair of an AAA is considered complex in cases of concomitant common iliac and/or IIAs, especially if they are bilateral and involve the iliac bifurcation, requiring revascularization of at least one IIA. Approximately, 10 to 20% of AAAs are combined with iliac aneurysms. Isolated iliac aneurysm accounts for 2 to 7% and isolated IIA aneurysms for 0.3 to 0.5% of all abdominal aneurysms. 1 2

An important guideline is the revascularization of at least one IIA, while the other one may be sacrificed. However, complications, such as buttock ischemia (including buttock claudication in 10–30%), erectile dysfunction, and bowel and spinal cord ischemia, may rarely develop, and are more frequent after bilateral IIA ligation. 3 Therefore, restoration of blood flow in one or both IIAs during repair of aortoiliac aneurysms or bilateral isolated iliac aneurysms is always desirable.

**Fig. 4:**
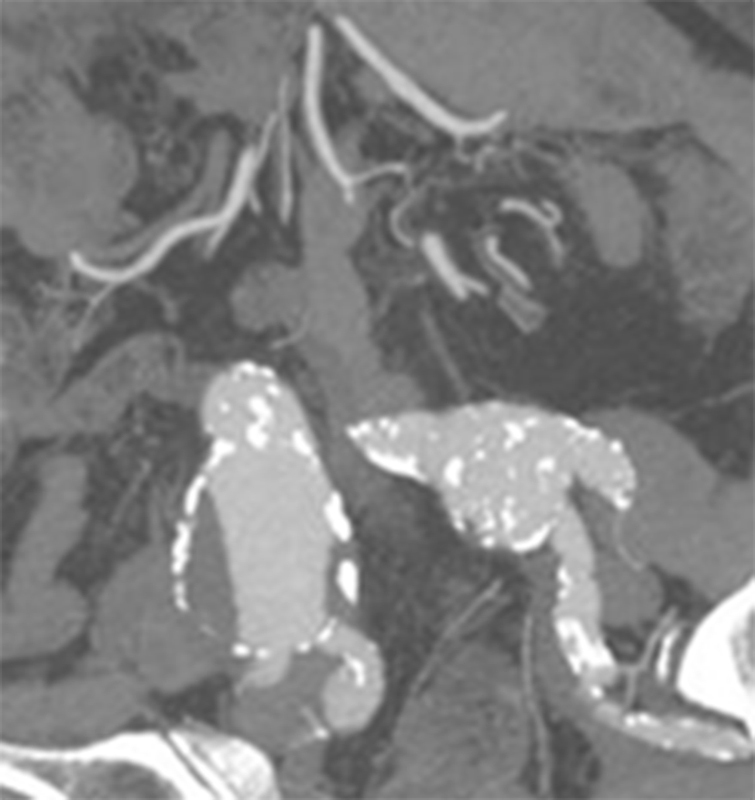
Computed tomography angiography showing the remarkable distance between the orifices of the internal iliac artery and external iliac artery in both sides.

In some cases, a formal distal end-to-end anastomosis of the graft limb with the common iliac bifurcation is impossible due to long distance between the orifices of the IIA and EIA, owing to aneurysmal dilatation of the distal common iliac artery ( Fig. 4 ). If this pathology is bilateral, there is need for reimplantation of the IIA in the graft limb (end-to-side fashion), or revascularization using a jump graft, after the completion of the end-to-end anastomosis of the graft limb with the EIA, at least on one side (the contralateral IIA can be ligated). 4 In comparison with this formal technique, the technique we describe herein may be performed more easily, it demands fewer anastomoses (four vs. five in case of repair with an aortoiliac graft anastomosed with the EIAs and revascularization of one IIA with a jump graft) and offers an alternative approach in emergency cases, if there is lack of the appropriate bifurcated graft due to logistic reasons.

Upon review of the literature, we found some alternative techniques that have been performed at least to one side (with contralateral IIA ligation) as follows:

- End-to-end anastomosis of the graft limb with the IIA end reimplantation of the EIA in the graft limb. 5 6 7
- Custom-made trifurcated graft which is tailored on table and provides a separate graft limb for revascularization of the IIA. 6
- A specific tube graft with a nitinol stent at one end (Gore hybrid vascular graft, GVHG; W. L. Gore and Associates Inc., Flagstaff, AZ). It is anastomosed centrally with the graft limb of a bifurcated graft, and is fixed after deployment of its stent-portion in the IIA distally. 1 It can be deployed over a 5-Fr Pruit catheter (LeMaitre Vascular, Burlington, MA) which is used to obtain distal control. This is important in cases of concomitant IIA aneurysms where the distal anastomosis may be time consuming and troublesome.
- The first AAA resection by Dubost et al 8 was performed in an AAA without iliac bifurcation involvement treated with interposition of a tube allograft between the aortic neck and the right common iliac artery. The contralateral iliac axis was dissected free and the thrombosed IIA was transected and ligated to bring up the endarterectomized common iliac artery for an end-to-side anastomosis with the tube graft. This approach could be followed in case of wide distance between the iliac orifices ipsilaterally, by insertion of a prosthetic tube graft from the aortic neck down to the iliac bifurcation. Then it is anastomosed in the same way with the contralateral iliac artery.

## Conclusion

In conclusion, depending on the aneurysm morphology and the difficulties arising in a specific operation, the surgeon decides if the modification, we describe here, can help to carry out the arterial reconstruction more easily.
